# Partial ectogestation and threats to the fetus: how healthcare professionals’ caution may reinforce the medicalization of pregnancy and childbirth

**DOI:** 10.1093/jlb/lsaf023

**Published:** 2025-10-28

**Authors:** Victoria Adkins

**Affiliations:** School of Law and Criminology, University of Greenwich, Old Royal Naval College, Park Row, London SE10 9LS, UK

**Keywords:** artificial placenta, caution, fetus, healthcare professionals, medicalization, partial ectogestation

## Abstract

Partial ectogestation is being developed in a bid to improve the survival rates and health outcomes associated with prematurity, but limited empirical research has been conducted on the views of key stakeholders, particularly healthcare professionals, in relation to this technology. This paper explores healthcare professionals’ perspectives in England on the use and implementation of partial ectogestation, within the medicalized context of pregnancy and childbirth. Following an online survey, qualitative interviews were undertaken with 22 healthcare professionals who work closely with pregnant individuals and fetuses. Using a formula of the precautionary principle from environmental studies, the analysis presented illustrates healthcare professionals’ apprehension toward partial ectogestation. With the fetus who may come to be transferred to an artificial placenta device at the centre of their concerns, participants were cautious of the technology producing poor outcomes and pushing the boundaries of nature. In response to these threats, they encourage strict criteria and clear parameters around the use of the technology. While healthcare professionals appear to endorse a social model of pregnancy when it comes to partial ectogestation, echoes of medicalization persist through medical determinations of poor outcomes and the continued centralization of the fetus as a patient.

## I. INTRODUCTION

The principal aim of developing medical technologies is generally to enhance care or improve upon outcomes currently being achieved. Partial ectogestation is a technological process whereby a fetus is delivered from a human placenta and placed in an artificial placenta device to continue its gestation outside of the human body. This technology is currently being developed with the primary aim of increasing the survival and morbidity rates associated with premature birth.^1^ While current neonatal intensive care has achieved success in recent years, with premature survival recorded at gestational ages as low as 22 weeks gestation, the care itself remains extremely intrusive. With the use of an artificial placenta, whereby an oxygenator pump is utilized and fetal circulation is sustained by means of umbilical cannulation, it is envisaged that the current strain of neonatal intensive care can be minimized as the premature lungs continue to develop as they would inside the human placenta.

While the success of artificial placenta devices is now widely recorded in animal studies^2^ and the Food and Drug Administration Paediatric Advisory Committee in the USA has undertaken discussions as to the future of the technology,^3^ as yet, there has been no application of the devices to human participants. Although partial ectogestation remains speculative in terms of clinical application, academic discussion of its potential implications has nonetheless escalated.^4^ However, very little empirical work, particularly in the UK, has been undertaken to discern whether partial ectogestation is desired or encouraged among its key stakeholders.^5^ A theoretical debate based on predicted attitudes and possible consequences of the technology may be ill-placed to direct any future regulation if it does not reflect the real views of key stakeholders. In seeking to address this gap and acknowledging that empirical research is ‘crucial in responsible innovation’,^6^ this paper presents analysis from the only known empirical study in England thus far that explores the perspectives of specific healthcare professionals in relation to partial ectogestation. Through the means of qualitative interviews, healthcare professionals, such as midwives and obstetricians, were asked to discuss their views on the uses and implementation of this new technology. The way in which healthcare professionals frame and approach partial ectogestation is of particular significance because of the authority that medicine holds within society^7^ and the moral legitimacy that healthcare professionals are considered to possess.^8^ Specific healthcare professionals are also likely to be confronted with the technology during clinical trials and may be responsible for guiding patients through their first interactions with it. Therefore, the views and attitudes of healthcare professionals toward partial ectogestation may influence and shape patient perspectives.

In what follows, this paper specifically focuses on one of three central themes,^9^ developed from the study’s analysis, titled ‘Proceed with caution’. This theme specifically focuses on the cautious approach participants wish to take toward partial ectogestation and the potential implications of such an approach. To structure and analyze the participants’ responses, a formula of the precautionary principle, borrowed from environmental studies, is used to illustrate the impetus behind their caution. While caution is well suited to a technology not yet tested in clinical trials, the analysis reveals a desire to minimize interference with human gestation, focusing on what is best for the fetus.^10^ Such a focus may be unsurprising from the medical community; however, the theme title ‘Proceed with caution’ also acts as a word of warning. If regulation comes to be based on what is best for the fetus, an opportunity arises for medical dominance over what are considered desirable outcomes and centralization of the fetus as the core patient persists. As will be discussed in the next section, the existing prevalence of risk and caution in pregnancy and childbirth has placed great pressure on pregnant individuals and caused a fragility to their autonomy. This paper therefore cautions against the assumption that healthcare professionals’ desire to restrict the use of partial ectogestation and to avoid interference in human pregnancy equates to an elevation of the status of pregnant individuals.

## II. BACKGROUND

### II.A. Risk and Caution in Pregnancy and Childbirth

Applying the precautionary formula to partial ectogestation is reflective of the general risk-averse approach toward pregnancy and childbirth. The avoidance of risk to the fetus is evident in the extensive guidance pregnant individuals are given including well-known lifestyle modifications such as dietary changes and the use of certain medications to less well-known behaviors such as the avoidance of changing cat litter.^11^ Additionally, while progress has been made with the inclusion of women in clinical research trials, pregnant individuals have remained largely underrepresented due to fears associated with adverse impacts on the fetus.^12^ This ironically means that many risk-averse behaviors promoted to pregnant people are not necessarily supported by evidence of harm.^13^ Risks are also experienced differently by different groups.^14^ In the UK, for example, reports indicate that women from Black ethnic minority and Asian backgrounds continue to be at higher risk of maternal death than White women.^15^ Structural racism also places more socioeconomic pressures on these groups, exposing them to levels of stress and making them more prone to risks of premature birth.^16^

The pervasiveness of risk has further been heightened by the medicalization of pregnancy and childbirth, the central lens through which the analysis in this paper is examined. While medicalization can be broadly understood to mean the way in which something is medically defined or framed, a sociological lens focuses on the way in which social issues have become redefined as medical problems.^17^ From a sociological perspective, the critique is that the medical profession has become the authority in determining how these problems should be understood and managed. This has resulted in a medical model of pregnancy, which promotes medical management and oversight and encourages preventative interventions.^18^ Risk to fetal health is placed at the center of how the process is managed,^19^ and, as a result, the fetus has come to be considered a patient,^20^ with intervention often encouraged when considered optimum for fetal wellbeing.

Although mortality rates have decreased over the past two decades and continue to do so,^21^ the risk culture comes at an autonomy cost to pregnant individuals. For example, Leshchenko and others argue that excluding pregnant people from clinical trials dismisses their role as advocates for themselves and the fetus.^22^ The reliance upon medical interpretations of ultrasounds and screenings also undermines the pregnant individuals knowledge of their own body^23^ and excessive oversight of the fetus arguably detracts from the care of the pregnant person.^24^ The minimized focus on the pregnant individual is also reflected in the contradictory relationship between risk and intervention. Lyerly and others, for example, highlight that small risks to the fetus associated with treatment for non-pregnancy-related illnesses or diseases often discourage intervention, despite how they may benefit the pregnant individual, and yet when it comes to birth, intervention is the immediate recourse.^25^

An increase in technology appears to have heightened the medicalization of pregnancy and childbirth,^26^ with the increased surveillance and intervention promoted as a means of identifying and reducing risks. The advent of partial ectogestation may then be considered as a further contribution to this approach, providing a further technological rescue of the fetus to avoid any risk they may face in the late stages of pregnancy.^27^

### II.B. Problems with Partial Ectogestation

While the desire to improve the survival and morbidity of premature ‘infants’ has been applauded, several concerns about the prospect of partial ectogestation have been raised. Firstly, the very fact that a placenta serves fetal needs and that partial ectogestation maintains fetal physiology has caused academic disagreement as to whether developers of the technology are right to refer to the entity undergoing the process as an infant or a neonate. Romanis, for example, uses the term ‘gestateling’ to differentiate the entity from a neonate and a fetus based on its limited interaction with the social world (by virtue of being sealed in the artificial placenta device) and its lacking biological adaptations (such as breathing through its lungs).^28^ Kingma and Finn further argue that the only characteristic the entity shares with a neonate is having changed its location from inside to outside the pregnant individual’s body.^29^ They consider the entity to have been ‘born-by-location-change’ but not ‘born-by-physiology-change’ and consider an artificial placenta to be supporting an entity that exhibits fetal physiology.^30^ Alternatively, Colgrove argues that the entity undergoing gestation in an artificial placenta is a different type of newborn, exhibiting life through a beating heart.^31^ Usuda similarly claims that there is nothing special about the entity undergoing partial ectogestation as the technology is simply another means of providing gas exchange.^32^ The views of healthcare professionals in this study toward how the entity within an artificial placenta is defined have been discussed elsewhere,^33^ but, ultimately, they too shared a lack of consensus on the point- with some considering the entity a neonate, others aligning it more so with a fetus and some recommending an ‘interim’ definition, similarly to that proposed by Romanis above. Crucially for this paper, regardless of how the entity comes to be defined once in the artificial placenta, the fetus subject to human pregnancy beforehand is centralized as the focus of concern.

In addition to definitional debates, academic discussion about the technology has itself been criticized for combining fears of ‘full’ ectogestation (the complete process of gestation taking place outside of the human body) with the present developing technology of partial ectogestation^34^ and it is often difficult to separate the critique of one from another. Nevertheless, anxieties about the future of the technology do play a role in consideration of its limited anticipated application.^35^ Of note is the concern that human pregnancy will become a ‘social anomaly’^36^ or that the rights of pregnant individuals will become diminished.^37^ While this appears to speak to a more advanced future of full ectogestation, the mere availability of an alternative form of gestation, even in a partial form, can elicit a protective stance toward human pregnancy. As this paper will demonstrate, however, a protective stance toward pregnancy does not necessarily equate to the protection of pregnant people’s rights and autonomy.

A further concern, and relevant to the participants' responses, is that while animal studies have been successful within their own parameters, there can be no absolute certainty that the success can be fully replicated in human participants.^38^ This is of particular concern when trying to decipher the long-term psychological impact that external gestation may have for the resulting infant.^39^ Singer and Wells have referred to this as a ‘catch-22’ situation whereby the adverse risks of ectogestation will not be known until the technology is applied, yet the risks may be too high for application to go ahead.^40^

### II.C. Applying the Precautionary Principle

Looking toward a sea of unknowns, it is not unusual for new innovations to be bound up with the possibility of risks and this habitually creates a sense of caution toward a technology’s application. In environmental studies, and reflected in healthcare policies, a precautionary principle is often applied to find a balance between over-zealous caution and innovation when implications or effects of a new development remain unknown.^41^ Sandin, in recognizing that the principle itself lacks guidance as to its application, draws upon four dimensions of the principle to formulate how it may be applied in particular scenarios.^42^ The formula reads:

‘*If* there is (1) a threat, which is (2) uncertain, *then* (3) some kind of action (4) is mandatory’ (emphasis in original).

Sandin’s formula, intended to direct decision-making in environment and health policy, is used in this analysis to illustrate healthcare professionals’ caution toward partial ectogestation. Partial ectogestation is yet to be trialed with humans, and there remains a lack of knowledge in relation to its clinical and social implications. The formula therefore helps to uncover what is underlying the caution of healthcare professionals—namely, the threat they consider partial ectogestation to pose. Firstly, the analysis will indicate how the unknown outcomes that partial ectogestation could produce are a cause of concern for healthcare professionals and, secondly, that partial ectogestation may encourage the crossing of particular boundaries. Both threats center around concern for the fetus (that may be about to be transferred to the artificial placenta) or the resulting child. In drawing upon the formula’s requirement to take action against such threats, the analysis outlines how participants propose limiting the use of partial ectogestation to last resort circumstances.

Since partial ectogestation is intricately connected to the processes of pregnancy and childbirth, attention must be paid to the medicalized and risk-averse context, referred to above, into which it may enter and how healthcare professionals’ framing of the technology may influence existing narratives around pregnancy. While partial ectogestation may be considered a further technological development that enhances medical control over gestation, participants in this study will be shown to use precaution to conversely justify minimizing interference with human gestation. Likely to endorse restrictive regulation of the use of artificial placenta devices, healthcare professionals appear to support use of the technology only in minimal last resort circumstances. The analysis in this paper therefore suggests that partial ectogestation may be used as an impetus toward a social model of pregnancy that encourages less intervention and allows nature to take its course.^43^ However, to determine when those limited circumstances justifying the use of an artificial placenta device arise, medical management and oversight inherent in the medical model will remain necessary. Further, the lack of intervention supported by participants in this study does not necessarily result in bringing the pregnant individual to the foreground as the core patient. Rather, the implementation of partial ectogestation will potentially provide a new fetal patient model that emphasizes closeness between the fetus and the pregnant individual by means of gestation.

## III. METHODS

The analysis discussed in this paper derives from a qualitative study exploring the views of specific healthcare professionals from National Health Service (NHS) Trusts in England in relation to the use and implementation of partial ectogestation. Healthcare professionals are considered a key stakeholder of partial ectogestation as they are likely to form a bridge between scientists/developers and the public, and medicine is considered to hold substantial authority within society.^44^ Further, legal management of abortion, for example, has been delegated to the authority of medicine^45^ and how healthcare professionals view partial ectogestation may influence how other stakeholders, such as pregnant individuals and future patients, approach and experience the technology.

An online survey was targeted toward healthcare professionals working in midwifery, obstetrics, gynecology, and reproductive and fetal medicine, as they work closely with both pregnant individuals and fetuses. A research poster was shared with 41 NHS Trusts in England who had confirmed capacity and capability, and the survey was open between June 2020 and December 2020. The survey was used to elicit preliminary views toward partial ectogestation to assist in the design of an interview guide and recruit for interviews. One hundred seventy-four full responses to the survey were received, and semi-structured interviews were carried out with 22 healthcare professionals between December 2020 and March 2021. Interview participants included 14 midwives, three consultant neonatologists, two consultant obstetricians and gynecologists, one consultant obstetrician, one antenatal and newborn screening coordinator, and one clinical nurse specialist. Interview participants were not linked to their survey responses, and it is the interview data that form the basis of the study’s analysis.

While survey distribution has the benefits of quick and widespread distribution, qualitative interviews allow a researcher to explore a topic in depth, thereby producing rich and descriptive data.^46^ The semi-structured nature of the interviews allowed for flexibility within and across the interviews while also ensuring participants did not stray too far from the research topic. This approach also allows participants to include additional thoughts, reflections, or related insights that are not necessarily in direct responses to interview questions, which often contribute to a more nuanced understanding of the topic. The interviews were initially designed to take place face to face, however in light of Covid-19, 21 of the 22 interviews took place on the telephone and one interview took place via Microsoft Teams (with audio only from the researcher for parity with the telephone interviews). Participant names have all been replaced with pseudonyms. Some participants selected their own pseudonyms, and, for those who did not provide their own, a gender-neutral name has been selected.

Participants were provided with an interview guide prior to the interviews, indicating that they would be asked to discuss their initial impressions of partial ectogestation, the potential advantages and disadvantages of the technology, and the future of the technology in terms of it being used beyond medical need. Within the interview guide participants were provided with a citation of the most relevant scientific paper at the time in relation to partial ectogenesis (as it was then referred to), with a quote from the paper outlining that the current aim of the technology is to improve outcomes ‘for those infants who are already being routinely resuscitated and cared for in neonatal intensive care units’.^47^ If pressed for further information in the interviews, the researcher would outline the functioning of the device in terms of the use of an oxygenator circuit and repeated the details from the paper that the lambs used in the animal studies were the equivalent of human fetuses at 22–24 weeks gestation. It should be noted that the research poster and survey described partial ectogenesis (the terminology in use at the time) as ‘the partial gestation of a foetus outside of the human womb’ and the interview guide included a quote from developers aiming to improve outcomes for ‘infants’ born prematurely. It could be argued that this presented participants with conflicting iterations of the technology. However, participants were asked about their own definitions of the entity that would be subject to partial ectogestation and analysis of these responses has been reported elsewhere.^48^ This study did not seek to support a specific iteration of the technology or definition of the entity. Rather, it drew on contemporaneous explanation of the technology and the stated aims of its developers. Importantly, participants were made aware of the ongoing debates regarding descriptions of both the technology and the entity and they were given space to present their own views,^49^ indicating that the technology was presented as accurately as possible at the time of the study. The unsettled nature of the descriptions of the technology, and in particular the outcomes it might produce, arguably contributed to the broader sense of uncertainty which underpins the theme explored in this paper.

Reflexive thematic analysis^50^ was applied to the interview data and is described as ‘a method for identifying, analysing and reporting patterns within data’.^51^ Braun and Clarke have developed their approach to thematic analysis since 2006, further outlining the theoretical assumptions underpinning their original publication.^52^ Reflexive thematic analysis centres researcher subjectivity, acknowledging the impact that the researcher has on the analysis of data.^53^ The researcher in this study, for example, took a data-driven approach, rejecting the use of a codebook or coding frame, which may be used in content analysis.^54^

Reflexive thematic analysis is underpinned by six recursive stages, which include familiarization and coding of the data. Familiarization, a process of immersion in the data, was undertaken during transcription and through continuous listening of the audio recordings and reading of transcripts. Coding requires tagging sections of the data that are considered meaningful to the research question, and the qualitative software package Nvivo 12 was used in this study to assist with organization of codes. Inductive coding was employed as no pre-existing framework was applied to the data.^55^ The coding process was data-driven with most codes being semantic in nature as they were generally descriptive and reflected the content of the extracted data.^56^ Initial themes were then generated by analyzing the relationship between codes and provisionally mapping patterns across the data. Rather than themes ‘emerging’ from the data, the generation of initial themes was undertaken by active choices being made by the researcher as to which codes relate to each other based on the researcher’s knowledge of the literature, the scope of the research, and their interests in the data.^57^ These initial themes were reviewed against the whole data set resulting in certain codes being moved or the parameters of themes being reshaped, thereby reflecting the recursive nature of the analysis process.^58^ The writing up of the themes played a substantial role in theme development as the themes took on much more depth and became ‘rich, contextualized stories’.^59^ At this stage, sub-themes were discarded to prevent analysis becoming fragmented and research questions were revisited to ensure the themes came together to tell a complete story about the data. The final analysis resulted in three key themes, one of which, ‘Proceed with caution’, is presented in this paper. The study received ethical approval from the Health Research Authority (IRAS: 270971) and Royal Holloway’s Ethics Committee (Project ID: 2086).

## IV. A NOTE ON TERMINOLOGY

It will be noted that the term partial ‘ectogestation’ is being used in this paper to reflect the updated literature that discusses appropriate terminology for the process of gestation in an artificial placenta.^60^ However, the terms partial ‘ectogenesis’ and artificial ‘womb’ were the language of the literature at the time the study was undertaken and may appear in some of the quotes of the participants. While Kingma and Finn align neonatal care with partial ‘ectogenesis’,^61^ both partial ectogestation ‘and’ partial ectogenesis, for the purpose of this paper, are considered to refer to the process whereby a fetus would be delivered from the pregnant individual and placed into an artificial placenta (formerly referred to as an artificial womb). This is to reflect that the discussion of partial ‘ectogenesis’ with participants at the time of the study correlates with what is now more accurately referred to as partial ‘ectogestation’. For readability, the word ‘technology’ is also used interchangeably with partial ectogestation (denoting it as a technological process).

## V. RESULTS

Sandin’s formula, above, which requires mandatory action in response to uncertain threats,^62^ is adopted to structure the results of this analysis in a bid to illustrate not only how the participants view partial ectogestation but also the way in which it may be introduced into clinical practice and regulated.

### V.A. Threat 1: Unknown Outcomes

When discussing both advantages and disadvantages of partial ectogestation, participants often referred to the uncertainty associated with the technology. This mirrored concerns in the literature regarding the unknown outcomes that partial ectogestation could produce^63^ and was fuelled not only by what was yet to be known about the technology itself, but also about pregnancy and childbirth. Devin and Rory, for example, made the following observations:

‘…I think a lot of childbirth is still a bit of a mystery and we still don’t even know really what causes women to go into labour’. (Devin, midwife)

‘…being pregnant and growing a baby is not just about being pregnant and growing a baby, there’s a lot more to it than that and a lot of things that we do not understand about how placental flow and how placenta’s work in the way that they provide the nutrition and non-nutritional support to the foetus as it is growing’. (Rory, Consultant Neonatologist)

Despite studies indicating how the intrauterine environment can impact the later development of the resulting child, such as neurological deficits,^64^ the continued lack of knowledge or ‘mystery’ of pregnancy and childbirth is acknowledged by the participants. Notwithstanding studies continuing to explore the risk factors associated with premature birth and potential causes,^65^ medical guidance suggests that there remains ‘no current test which accurately predicts preterm delivery’.^66^ In addition, the World Health Organization continues to call for further research into the causes and mechanisms of preterm birth.^67^ This knowledge gap conceded by the participants caused them to question the potential success of partial ectogestation:

‘…So, potentially, theoretically, you could possibly do it to a more simple creature, but I’m not convinced you’d be able to do it with a human without potentially significant impacts on development and psychological development’. (Rory, Consultant Neonatologist)

Confidence in the ability of partial ectogestation to imitate human gestation is therefore stifled if the process to which the technology seeks to imitate (gestation) is not fully understood. Sander-Staudt has similarly outlined concerns surrounding what we do not know about the fetal experience of the womb and how this makes it difficult to know what impacts a form of artificial gestation may have.^68^ The womb itself has also been described as an organ of mystery, enhancing concerns around the disruption of the human connection in pregnancy.^69^ Sacha, again thinking about when partial ectogestation may be used, made particular reference to impacts on the development of the gestated entity:

‘…For me, I’d want to be clear about what the benefits of the technology were. So if what could be proven was that there was a reduced amount of physical or neurological disability was reduced by using the technology for premature babies, then I’d be all for it. But if it’s just a case of “well we can grow a baby outside the womb but we don’t know that it improves neurological or disability outcomes in the long term”, then I think I’d be saying well why are we then doing this? If we can’t improve the outcomes then why are we doing it?’ (sic) (Sacha, Midwife)

The promotion of the technology, in Sacha’s view, is very much dependent on the outcomes it produces. If outcomes, such as reduced disability, are achieved, then the purpose of partial ectogestation is considered fruitful. However, an inability to be confident in these outcomes due to a lack of knowledge was a recurring concern:

‘Again, I think the fact that at the moment there is no mechanism for a baby being grown outside of a womb. We don’t know if that, potentially, would have detrimental effects mentally or physically’. (Innis, Midwife)

Again, Innis, in thinking about potential disadvantages of partial ectogestation, reflected on the fact that partial gestation via artificial placentas has not yet been shown to produce non-detrimental outcomes for human infants. As Hammond-Browning has noted, human gestation is currently the only proven successful method of reproduction and therefore comparison with an artificial placenta is not possible.^70^ Reflecting this, Leia, a consultant neonatologist, commented that ‘…a huge amount of research will be needed before we can even begin to understand what sort of long-term effects this will have on the fetus, who then becomes a baby’. The participants therefore found themselves in the catch-22 situation discussed above whereby the desire for the knowledge of successful outcomes can only come about by applying the technology.^71^ Yet it is precisely the existing lack of knowledge that made participants cautious toward its implementation.

The participants’ caution toward partial ectogestation, fueled by an uncertainty of the outcomes it could produce, is not surprising and provides a contrast to the academic discussion of the technology, which ‘encourages readers to think of the future with little regard for what is required to get there’.^72^ The participants’ concern with the purpose of the technology adds a new perspective to the discussion as they concentrated on what it is that the technology is attempting to achieve. Blake, for example, when discussing the translation of the technology to humans, reflected on current survival rates of premature babies:

‘… I mean they’re resuscitating babies at 23 weeks now and they’re getting okay outcomes. But I think what we think of, I think what I think as an okay outcome, which is a completely normal educational achievement of a child, is perhaps not actually what they’re achieving. Simplistically the achievement is much lower than that. The bar is set much lower’. (Blake, Consultant Obstetrician and Gynecologist)

Similarly, Frances asked:

‘…you sort of think, even though it’s reduced to 24 what’s the quality of life for the babies? You get good statistics, neonatal units, babies discharged, brilliant, but, and I don’t know the figures as to how many of those babies are on long-term oxygen at home, how many are on long-term tube feeding?’ (Frances, Antenatal and Newborn Screening Coordinator)

When considering the use and potential disadvantages of partial ectogestation, participants were therefore hesitant to place an inordinate focus on ‘survival’ and wanted to look more closely at what the reality of that survival looks like.^73^ Neonatologists have similarly been found to feel that technology is sometimes overused as an ‘heroic means of extraordinary support’ for the treatment of extremely premature infants.^74^ Accoe and others have also noted that the moral defensibility of preventing neonatal death through partial ectogestation may cause the quality of life that results to be overlooked.^75^ The healthcare professionals in this study appear to caution against the success of partial ectogestation being pinned simply on the survival of the resulting child, connecting to the earlier desire for meaningful outcomes.

The unknown outcomes that partial ectogestation may produce therefore constitutes an uncertain threat in accordance with Sandin’s formula. The uncertainty is sourced not only from the technology’s lack of application to humans but also from the ‘mystery’ that continues to shroud pregnancy and childbirth. The outcome-based focus of the participants aligns with their own professions which target efforts toward the sustained health and wellbeing of both pregnant individuals and their fetus/resulting child. The desire for the technology to produce meaningful outcomes to justify its purpose however falls victim to Singer and Wells’ catch-22 dilemma. As with most developments, research of the long-term effects of partial ectogestation will only provide the required knowledge after the technology has been applied.^76^ As an example, studies confirming that children born from in-vitro fertilization are no less psychologically disadvantaged than adopted children, or those conceived naturally, have lagged far behind the technology itself.^77^ Therefore, in order to reach those meaningful outcomes, some less desirable results may need to occur as the technology is trialed and applied to human participants.^78^ The question as to what outcomes are considered meaningful or desired is discussed later in the paper.

### V.B. Threat 2: Pushing ‘Natural’ Boundaries

When asked about the potential disadvantages of partial ectogestation, participants also allured to concerns around a temptation to keep unhealthy fetuses alive. Lucy for example, wondered whether:

‘…boundaries would be pushed to keep foetuses alive that maybe are potentially already damaged and maybe wouldn’t have a very good quality of life…There’d be some sort of moral imperative to keep them alive at the expense of their own quality of life I suppose’. (Lucy, Midwife)

Like the earlier threat outlined, Lucy remained concerned with undesirable outcomes. However, partial ectogestation was being viewed, in this instance, as a tool to maintain a fetus that is already known to be unhealthy. The use of the technology in this circumstance was considered to be crossing a particular ‘boundary’. Using the example of a quadriplegic child, Blake fleshed out a similar concern in more depth:

‘The big risk is that this baby may have, should have died in some ways. If we are not going to save its life…And maybe there is an element of maybe that child should never have been, not born, but never have survived…Should we have done that because we can or should we have said actually no it’s not fair on the child…So that’s the bit I think that’s the downside’. (Blake, Consultant Obstetrician and Gynecologist)

Similar to Lucy’s anxiety of partial ectogestation being used in circumstances when a poor quality of life is known to result, Blake appeared to allude to an apprehension about the technology interfering with a particular course of events. This strikes a resemblance with the adage of allowing nature to take its course and specific references to nature were made by the participants themselves. For example, Frances, in thinking about disadvantages of partial ectogestation, reflected upon premature babies being kept alive and still being born with severe disabilities and asked:

‘And then you think, “Oh, was that such a great idea, sometimes to fight nature so hard?”’ (Frances, Antenatal and Newborn Screening Coordinator)

The concept of ‘fighting nature’ depicts partial ectogestation as being at odds with the natural course of events that should have perhaps been allowed to prevail. The contention between medicine and nature is long-standing; however, it is not always natural processes, particularly in reproduction, that have been favored. Oakley, for example, described the fear among obstetricians in the seventies and eighties of the ‘unknown laws’ of nature that could undermine ‘human expertise’.^79^ The participants, however, seemed to suggest that medicine and/or technology have interfered too readily in circumstances where nature should have taken its course.

The Roman Catholic Church has similarly depicted in vitro fertilization as a form of interference due to its disruption of natural procreation and the development of embryos,^80^ and nature has continued to become personified, in the way expressed by Frances above, as assisted reproductive technologies have developed. The Nuffield Council on Bioethics, for example, has reflected on discourses where nature is considered to have its own knowledge about what should and should not happen.^81^ Despite the participants’ earlier admission as to what is still unknown about pregnancy and childbirth, human gestation has nevertheless proved itself capable of producing favorable outcomes for fetuses. Student midwives in the UK are also found to oppose the excessive use of technology in reproduction,^82^ and midwives, who make up a large proportion of the participants in this study, are known to associate themselves with a philosophy of framing pregnancy as a natural physiological process.^83^ For the participants in this study therefore, it may be that nature is considered to hold an authoritative source of knowledge. This therefore justifies more trust being put into the ‘natural’ process of human gestation rather than partial ectogestation.

### V.C. Taking Action

The use of Sandin’s formula in this analysis thus far has resulted in two potential ‘threats’ of partial ectogestation. There is the potential of the technology to produce unfavorable outcomes for the fetus or resulting child and there is the possibility of it interfering too readily with human gestation (which may also produce a less favorable outcome, even compared to death). The remainder of Sandin’s formula requires that in response to these uncertain threats ‘…some kind of action…is mandatory’. Forms of action were apparent within the participants’ responses, particularly when discussing the circumstances in which they considered partial ectogestation would be used:

‘…perfect for those women who get severe preeclampsia and they’ve got a very hostile intrauterine environment and therefore you can provide an extrauterine environment that is as best as we can make it’. (Harley, Consultant Neonatologist)

‘I would think that the scenario where this would be useful would be in those women who medically are not able to continue with pregnancies for various reasons or those perhaps who have actually developed infection or who’ve got cervical incompetence where the pregnancy is going to be lost earlier on’. (Quin, Consultant Obstetrician and Gynecologist)

In these scenarios, the participants imagined partial ectogestation being utilized when human gestation is no longer possible or too high risk. The intrauterine environment must be ‘hostile’ and unsustainable before the technology is put into use. Partial ectogestation is therefore something to be used when an emergency has struck and to be relied upon as a last resort:

‘So, in the sense of, it’s strictly for people who have no choice. You know, they are in that situation, and that is, kind of, the last option’. (Frances, Antenatal and Newborn Screening Coordinator)

The reference to ‘no choice’ and last options suggests that partial ectogestation is engaged with when all other treatment options have been exhausted. Similarly, Francesca, a midwife, commented that the technology would be used in ‘emergency situations’ where an infant is unlikely to survive without this ‘emergency input’. Participants therefore appear to place clear parameters around the use of the technology and this form of action limits its use only to those circumstances where human gestation is not sustainable.

The limited scope to apply the technology was also evidence of participants wishing to maintain human gestation as much as possible and thereby reduce the interference of partial ectogestation:

‘And we have women who have severe pelvic pain or they have conditions that mean carrying a pregnancy is physically very demanding for them. However, we have physiotherapists, we have painkillers, we have support for them. It’s not ideal and the pregnancy will be very challenging for them but I wouldn’t, I would still think that in utero pregnancy is the best. I don’t think we should be swapping for an external option when we can continue a pregnancy in utero’. (Ash, Consultant Obstetrician)

Here, we can see a clearer indication from Ash of the ways in which other resources would be called upon to maintain human gestation prior to partial ectogestation being considered. Devin also illustrated the high threshold that would be put in place to justify the use of the technology:

‘I think it would have to be a very serious reason. We occasionally get women with very severe pre-eclampsia and we’re having to deliver at 32 weeks or so. So, I think if that was the case or say she had a cardiac condition or a liver condition or something that limits her capacity to be able to grow a baby further, then I think in that situation it’s fine. I think for something like backache, no’. (Devin, Midwife)

Again, it is those desperate and severe last resort scenarios where the use of partial ectogestation would be acceptable with efforts focused on maintaining human gestation until such ‘very serious reasons’ arise. This stands in stark contrast to those who suggest that the technology could be used to alleviate the strains of pregnancy^84^ to reflect the cure over management approach to medicine.^85^ However, the participants quite clearly indicated that they would rather maintain human gestation as much as possible before introducing partial ectogestation. One of the participants articulated this position succinctly:

‘…nature is ultimately better. This should never replace nature. But, if we’re using it in and alongside nature to help save babies where nature can’t save then, then I think it’s okay’. (Blake, Consultant Obstetrician and Gynecologist)

This contrast between partial ectogestation replacing nature and being used alongside it clearly demonstrates how the technology is not considered as a tool to overcome human gestation, but merely as an aid when human gestation cannot be maintained.

Following Sandin’s formula, the participants’ mandatory action against the perceived threats of partial ectogestation would be to place a high threshold on justifying its use and to limit such use to last resort scenarios. It therefore appears that minimizing the interference with human gestation would be a goal of healthcare professionals. An approach of non-interference is described by Singer and Wells as a descriptive view of nature—a nature that is untouched by human intervention.^86^ However, an endorsement of this by healthcare professionals would seemingly result in very little reproductive healthcare being provided at all and medicine in many respects aims to prevent natural events within the body.^87^ An alternative approach, known as the teleological view, regards the interference of human nature to be a result of nature itself. Human interference, including the production of technologies, is a natural evolution of human capabilities^88^ and would align with the development of medicines and technologies that seek to improve outcomes. Mercurio, for example, has suggested that artificial placentas may in fact be better aligned to the natural physiological process of gestation than standard neonatal care.^89^

From the analysis, the participants allow for partial ectogestation to interfere with human gestation to some degree, albeit in minimal last resort circumstances, and this may be explained by Norman’s theory of the ‘threshold effect’.^90^ According to Norman, a threshold effect is put in place when deciding how ‘far’ human action should interfere with nature. On this account, background conditions that are usually taken as a ‘given’, such as human gestation in this context, should remain untouched for fear that interference may result in a lack of meaning and value being attached to that condition. This approach suggests that participants were seeking to uphold the value of human gestation by limiting interference with it.

This value was alluded to by participants when discussing the disadvantages of partial ectogestation for the fetus. For example, Mary stated:

‘There’s a really important aspect, physically, emotionally, psychologically, that is scientifically shown now in neurological research and things, about women talking to their babies in utero, bonding with them, and the ability for these children to form attachments when they’re older’. (Mary, Midwife)

Similarly, in discussing whether partial ectogestation could ever be proven as safe as human gestation, Tate expressed:

‘I think what would be really interesting is looking at the bonding in particular between the pregnant individual and the foetus and subsequent baby long term, because there is so much feedback between the foetus and the pregnant individual when the baby is in the womb’. (Tate, Midwife)

The desire of participants to minimize interference with human gestation may therefore be linked to a desire to maintain the intimate relationship between the fetus and the pregnant individual. Pat, a midwife, further questioned that if the fetus is removed from the pregnant individual during gestation ‘…who is going to give it that sound, that touch, all of those unconscious things, that help them with their brain development and everything before they’re even born?’. The removal of these ‘unconscious things’ suggests that the fetus may be deprived of something if removed from human gestation and again we cannot be certain of what those things are in light of their ‘unconscious’ nature.

The relationship between the fetus and the pregnant individual that participants valued can be linked back to their earlier lack of confidence in partial ectogestation. When considering whether partial ectogestation could be as good as a human placenta, Lucy commented:

‘There’s that symbiotic relationship when they are inside each other that an artificial foetus would not have. So, it’s lacking things that probably isn’t, in a way, in its own artificial environment that probably can’t be replicated’. (Lucy, Midwife)

Similarly, Tate expressed:

‘They’re listening to voices, the tone of people’s voice, the touch, just the moving about as well, because, the movements of the mother can trigger the movements of the foetus so it’ll be interesting in terms of how on earth they build an artificial womb that mimics that much of the pregnant individual’. (Tate, Midwife)

The belief that human gestation provides something for the fetus that cannot be replicated in an artificial placenta may therefore explain why participants sought to minimize disruption to it. An emphasis on intrauterine bonding however may prove detrimental to those engaged in surrogacy arrangements or primary carers of the child who have not gestated.^91^ It is no longer a given that the individual gestating will become the primary carer of the child or even have any biological relation to them. Non-gestating partners and intended parents in surrogacy arrangements have been shown to use ‘imaginative identification’ and other practices to create bonds with the fetus^92^ and a focus on intrauterine bonding may undermine these alternative forms of creating connection. Those developing artificial placenta devices are also exploring adding features such as maternal heartbeats and abdominal sounds.^93^ Therefore, the current imaginative practices undertaken by non-gestating partners may be adopted to reduce the disruption to gestational bonding when partial ectogestation is used.

## VI. DISCUSSION

Application of Sandin’s formula to healthcare professionals’ views on the use and application of partial ectogestation indicates two threats they considered the technology to pose— firstly, its potential of producing unknown outcomes for the fetus, and secondly, the prospect of it crossing boundaries and thereby interfering with human gestation. The action they propose in response to these threats is to impose strict criteria and parameters around the use of the technology. The following discussion will explore the implications of this analysis through the lens of the medicalized nature of pregnancy and childbirth. However, before doing so, it is worth noting that concern for the welfare of the child by healthcare professionals in this study is not necessarily surprising or misplaced as their professional roles place emphasis on upholding the health and wellbeing of their patients, which often includes the fetus. Although midwives are commonly associated with a social model of pregnancy, maternity practice is argued to encompass a mixture of both social and medical models of pregnancy along a continuum and midwives are limited by clinical guidelines which often follow a medical model.^94^ In addition, the Human Fertilisation and Embryology Act 1990 (as amended) requires clinicians to consider the welfare of any future child when providing treatment services (such as in vitro fertilization) under the Act,^95^ and even those who advocate for partial ectogestation on the basis of reproductive autonomy do so with the caveat that the technology is proven safe for the fetus/resulting child.^96^ Arguably the remaining conflict between a social and medical model of pregnancy derives from the advancement of medical technologies and legislative endorsement. Nevertheless, the medical model provokes criticism surrounding medical dominance and the undermining of reproductive autonomy of pregnant individuals. As the following discussion will demonstrate, relying exclusively on healthcare professionals’ perspectives to shape regulation of partial ectogestation risks perpetuating these criticisms, highlighting the need to consider the collective views of all stakeholders.

### VI.A. Discouraging Interference

The analysis presented in this paper illustrates that the way in which participants framed partial ectogestation was highly dependent on the outcome that the technology produces. If the technology is successful in the last resort scenarios that participants allow (by producing a better than predicted outcome), it is an aid to human gestation. Alternatively, if the technology is used outside of these circumstances and/or produces a poor outcome, it is an interference and ‘fighting nature’. These contradicting interpretations reflect the distinction often made between ‘assisted’ reproductive technologies and ‘artificial’ reproductive technologies.^97^ The former assists nature, allowing it to get ‘back on track’^98^ much like partial ectogestation would do in those last resort scenarios; however, technology is artificial and at odds with nature when it is viewed as seeking to take control over natural processes. The conflicting representation of partial ectogestation is also mirrored in academic debates, with the technology often heralded for its potential to revolutionize the care of premature infants,^99^ alongside warnings of its ability to undermine the capabilities of the human body.^100^

The participants’ skepticism of the outcomes partial ectogestation may produce led to their desire to limit its interference with human gestation. This would seemingly suggest an endorsement of the social model of pregnancy, which upholds the ability of the human body to undertake the physiological process of gestation. In defining limited circumstances in which partial ectogestation can be applied, human gestation is largely allowed to run its course without intervention. On this basis, healthcare professionals are likely to support the introduction of partial ectogestation into the clinical domain in a very restricted and limited capacity. Regulation of the technology, from the perspective of healthcare professionals, would likely be risk-averse with support of a public ordering form of regulation whereby legislation governs how the technology is used.^101^ It may be reflective of the ‘principle but pragmatic, strict but permissive’ stance taken with embryo research^102^ which reflects healthcare professionals’ (dis)trust in the technology balanced with their desire to provide benefit in last resort scenarios. This restricted use of partial ectogestation may encourage a less interventionist and therefore arguably less medicalized approach to pregnancy.

However, the healthcare professionals’ approach is not entirely detached from a medical model. That fetal wellbeing underpins the ‘threats’ they associate with partial ectogestation can be linked to the concept of risk that governs much of the medicalization of pregnancy and childbirth. Many pregnancies are now punctuated with regular check-ups and antenatal appointments, and several births often take place in hospital settings in order to manage fetal health.^103^ Restricting the use of partial ectogestation is unlikely to diminish the monitoring and oversight inherent in pregnancy management and, in fact, observation will likely be necessary in order to determine when the use of an artificial placenta device may be justified.

Beyond the maintenance of medical oversight, however, the desire to avoid risks or poor outcomes for a fetus who may be transferred to the device raises the question as to what constitutes a poor outcome. The medicalized nature of pregnancy and childbirth has often left pregnant individuals relying on healthcare professionals to tell them whether their pregnancies are progressing in the ‘right’ way^104^ and the same can be said when it comes to making decisions around the outcomes of those pregnancies. For example, the availability of prenatal screening and the disabilities screened for can send messages about the kinds of lives that are worth living or the kind of child that should be desired.^105^ Using partial ectogestation to avoid the comorbidities associated with premature birth may be considered as a further example of enabling ‘eugenic logic’,^106^ surrounding desires to eliminate disability. Healthcare professionals’ support for limited use of the technology might then suggest a desire to move away from this; however, some participants’ responses indicated otherwise. For them, the desire to allow nature to run its course and for human gestation not to be interfered with was rooted in the belief that partial ectogestation could otherwise keep alive a fetus that ‘should have died’. Fears that partial ectogestation would cross boundaries in this regard was therefore driven by a desire to still avoid a poor outcome. Inherent within these responses were arguably value judgments as to an acceptable or desirable quality of life,^107^ and certain examples were alluded to such as long-term reliance on oxygen and quadriplegia. Medical definitions of disability are argued to underpin social and political framings^108^ and healthcare professionals’ perspectives on what constitutes a poor outcome from the use of partial ectogestation could further contribute to these narratives. Accoe and others warn that a focus on reducing morbidities may ‘reinforce a bias in favour of “normalcy”’.^109^ While studies suggest that clinical outcomes are not the only factor that parents consider when deciding whether to proceed with a pregnancy following prenatal testing,^110^ the moral legitimacy afforded to the healthcare profession adds moral weight to the diagnostic tools offered and can therefore shape the decisions of prospective parents.^111^ Policy decisions that determine when and why partial ectogestation can or cannot be used have the potential, therefore, to direct pregnant individuals’ behaviors and decision-making about the use of the technology.

Definitive criteria as to when partial ectogestation should be used were not fully established in this study, and further work needs to be undertaken to understand how desirable or undesirable outcomes are defined.^112^ The healthcare professionals in this study were inordinately focused on the implications of partial ectogestation on the fetus and unsurprisingly placed emphasis on clinical outcomes. To base regulation on this approach, however, would emphasize the dominance of medical framings of ‘good’ pregnancies and fail to account for how outcomes are viewed and experienced beyond clinical parameters.^113^ In relation to partial ectogestation, specifically, calls have been made to move away from fetal centricity and account for how the technology may impact the pregnant individual.^114^ Not only would they be making a decision about the future of their fetus, but those decisions would also impact the trajectory of their pregnancies.^115^ If an outcome-based approach as taken by the healthcare professionals is to inform future regulation, it needs to be further informed by other stakeholders.

### VI.B. Fetal-Patient Narratives

Further caution also needs to be applied to the way in which healthcare professionals position the fetus at the center of their outcomes-based concerns. The prioritization of the fetus in the medical model of pregnancy is often criticized for placing fetal needs above those of the pregnant individual,^116^ with the fetus considered as a patient.^117^ The concept of the fetal patient is also supported by the development of fetal medicine^118^ and is argued to derive from healthcare professionals’ beneficence-based obligations.^119^ The idea that the fetus is now a patient has further led to it being framed as an entity separate from the pregnant individual.^120^ The medicalization of pregnancy and childbirth has therefore resulted in a fetal-patient-separation model where the needs of the fetus often require intervention that results in the fetus being separated from the pregnant individual. Partial ectogestation has already been depicted as a device that could fulfill this separation in real terms by rescuing the fetus either from an unfavorable intrauterine environment^121^ or from the pregnant individual when they may be considering a termination of the pregnancy.^122^ There are concerns therefore that the reproductive autonomy of pregnant individuals could be subverted with them being encouraged to relinquish their fetus to the technology.^123^

In contrast, healthcare professionals in this study were cautious about partial ectogestation intervening with human gestation and endorsed its minimal use. Nevertheless, they continued to place the fetus at the centre of their considerations and perpetuate the perspective of the fetus as a patient. A fetal-patient narrative therefore prevails; however, the rejection of intervention with partial ectogestation creates an alternative fetal-patient-closeness model. Within this model, the fetus is still featured as a patient but its needs demand that it remains with(in) the pregnant individual rather than be separated from them. As human gestation is the only current proven safe method of gestation,^124^ maintaining the closeness between the fetus and the pregnant individual remains necessary for the healthcare professionals when compared to the unknowns of partial ectogestation. Where the original fetal-patient-separation model is feared to rely on a pathologizing of the female body^125^ with human gestation considered inferior, the fetal-patient-closeness model encourages human gestation to be considered superior because it is known to be able to produce ‘good’ outcomes. This reframing of the fetal-patient narrative, drawn from healthcare professionals’ perspectives on partial ectogestation, reinforces rather than dismantles the perceived primacy of human gestation.

Despite a reframed model, the issue remains that it is not the individual undertaking the gestation that is being centred by healthcare professionals, notwithstanding their reflections on the difficulties experienced during human gestation.^126^ With a primary focus on the wellbeing of the fetus, the risk remains that the interests of the pregnant individual are overlooked.^127^ In both a separation and closeness model, a pregnant individual is expected to do what is best for the fetus,^128^ whether this means allowing the fetus to be separated from them or maintaining a pregnancy, both of which may be against their own interests. It is also an undermining of the individuality of pregnant individuals to assume that their interests will always align with what is best for the fetus.^129^ Restrictive regulation of partial ectogestation to reduce its intervention may therefore contribute to existing critiques of the medical model of pregnancy. Placing fetal needs at the forefront of regulation may undermine the autonomy of pregnant individuals and ignore that their needs and the needs of the fetus are interdependent.^130^ Although the development of partial ectogestation is dedicated toward improving health and wellbeing statistics surrounding premature birth, this may not be appropriate as the sole basis of restrictive regulation. It is important to remember that the pregnant individual is as much a part of the intrauterine relationship that the fetal-patient-closeness model endorses, and therefore, their needs and desires should feature in considerations of outcomes and regulation of the technology.

While the precautionary principle and its structural formula designed by Sandin has been useful in analyzing healthcare professionals’ approach to partial ectogestation, the precautionary principle has been criticized for promoting the prohibition of all harm^131^ and, as such, has been promoted as one principle to be weighed against others, rather than being treated as absolute.^132^ What it has shed further light on this study, however, is how precaution can narrow attention to risk avoidance at the expense of broader factors and the voices of other stakeholders.

## VII. CONCLUSION

This paper presents analysis from one of the first studies in England to consider the views of healthcare professionals in relation to partial ectogestation. The views of healthcare professionals regarding partial ectogestation are important as they may have responsibility for introducing the technology to patients and can shape public perspectives.

Several significant points are raised by the analysis presented in this paper. The cautious approach of the participants toward partial ectogestation suggests that healthcare professionals are most likely to support its use in limited circumstances and, supporting calls for a more realistic discussion of the technology, the participants endorse a detailed focus on the outcomes produced by the technology that look beyond mere survival of the fetus or resulting child. Regulation from the perspective of healthcare professionals should therefore be restrictive rather than permissive. However, while healthcare professionals’ caution toward intervention suggests an endorsement of the social model of pregnancy, medical management will be necessary to determine when the technology may be used. In addition, characteristics of medical dominance still permeate if healthcare professionals alone are left to determine what constitutes a poor outcome when using an artificial placenta.

In addition, a centralizing of the fetus in determining how and when partial ectogestation should be used may result in a fetal-patient-closeness model whereby the needs of the fetus require that it remains with(in) the pregnant individual. This has the potential to perpetuate the sociological critique of the medicalization of pregnancy and childbirth, in that inadequate regard is given to the needs and desires of the pregnant individual when managing a pregnancy. Although healthcare professionals may be well placed in their caution and support of a restrictive form of regulation toward partial ectogestation, a too-narrow approach may not take sufficient account of pregnant individuals, without whom natural gestation would not be possible. Therefore, future regulations governing the use of partial ectogestation should be guided by the collective perspectives of all stakeholders, ensuring that medical considerations do not unduly dominate decision-making.

## Footnotes

1 Emily A. Partridge, Marcus G. Davey, Matthew A. Hornick, Patrick E. McGovern, Ali Y. Mejaddam, Jesse D. Vrecenak, Carmen Mesas-Burgos *et al*., *An Extra-Uterine System to Physiologically Support the Extreme Premature Lamb*, 8 Nat. Commun. 15,112, 15,112 (2017); Felix R. De Bie, Marcus G. Davey, Abby C. Larson, Jan Deprest, and Alan W. Flake., *Artificial Placenta and Womb Technology: Past, Current, and Future Challenges towards Clinical Translation*, 41 Prenatal Diag. 145, at 158 (2021).

2
*Id.*; Haruo Usuda, Shimpei Watanabe, Yuichiro Miura, Masatoshi Saito, Gabrielle C. Musk, Judith Rittenschober-Böhm, Hideyuki Ikeda *et al*., *Successful Maintenance of Key Physiological Parameters in Preterm Lambs Treated with Ex Vivo Uterine Environment Therapy for a Period of 1 Week,* 217.4 Am. J. Obstet. Gynecol. 457.e1 (2017); Haruo Usuda, Shimpei Watanabe, Masatoshi Saito, Shinichi Sato, Gabrielle C. Musk, Ms Erin Fee, Sean Carter *et al., Successful Use of an Artificial Placenta to Support Extremely Preterm Ovine Fetuses at the Border of Viability,* 221.1 Am. J. Obstet. Gynecol 69-e1; Matther A. Hornick, Ali Y. Mejaddam, Patrick E. McGovern, Grace Hwang, Jiancheng Han, William H. Peranteau, Emily A. Partridge, Marcus G. Davey, and Alan W. Flake, *Technical Feasibility of Umbilical Cannulation in Midgestation Lambs Supported by the EXTra-uterine Environment for Neonatal Development (EXTEND),* 43 Artif. Organs 1154 (2019).

3 US FDA, *Pediatric Advisory Committee Meeting Announcement,*  https://www.fda.gov/advisory-committees/advisory-committee-calendar/pediatric-advisory-committee-meeting-announcement-09192023 (accessed July 30, 2024).

4 Elizabeth Chloe Romanis, *Artificial Womb Technology and the Frontiers of Human Reproduction: Conceptual Differences and Potential Implications*, 44 J. Med. Ethics 751 (2018); Elizabeth Chloe Romanis, *Artificial Womb Technology and the Choice to Gestate Ex Utero: Is Partial Ectogenesis the Business of the Criminal Law?,* 28 Med. Law. Rev. 342 (2020); Natasha Hammond-Browning, *A New Dawn: Ectogenesis, Future Children and Reproductive Choice*, 14 Contemp. Iss. Law 349 (2018); Seppe Segers, Guido Pennings and Heidi Mertes, *The Ethics of Ectogenesis-Aided Foetal Treatment* 34.4 Bioethics 364, at 368–369 (2020); Victoria Adkins, *Impact of Ectogenesis on the Medicalisation of Pregnancy and Childbirth*, 47.4 J. Med. Ethics. 239 (2021).

5 For exceptions in the UK, see Elizabeth Chloe Romanis, *Equality-Enhancing Potential of Novel Forms of Assisted Gestation: Perspectives of Reproductive Rights Advocates*, 37.7 Bioethics 637 (2023); Elizabeth Chloe Romanis, *‘The Law Is Very, Very Outdated and Not Keeping Up with the Technology’: Novel Forms of Assisted Gestation, Legal Challenges, and Perspectives of Reproductive Rights Advocates in England and Wales*, 10.2 J. Law Biosci. lsad027 (2023). Outside of the UK, see Angret de Boer, Angret, André Krom, Rania Kalaai, Marieke de Vries, Marije Hogeveen, Sylvia A. Obermann-Borst, Marijn Vermeulen *et al*., *Stakeholder Perspectives on the Design of First-In-Human Trials for Artificial Amnion and Placenta Technology: A Qualitative Study*, Bjog 10 (2025); Leslie Cannold, *Women, Ectogenesis and Ethical Theory,* 12.1 J. Appl. Philos. 55 (1995); Frida Simonstein and Michal Mashiach-Eizenberg, *A Survey of People’s Attitude Towards the Artificial Womb and Ectogenesis in Israel* in Reprogen-Ethics And The Future Of Gender (F. Simonstein, ed, 2009); Lydia Di Stefano, Catherine Mills, Andrew Watkins, and Dominic Wilkinson, *Ectogestation Ethics: The Implications of Artificially Extending Gestation for Viability, Newborn Resuscitation and Abortion*, 34.4 Bioethics 371 (2020).

6 André Krom, Angret de Boer, Rosa Geurtzen, and Martine C. de Vries, *Capabilities and Stakeholders–Two Ways of Enriching the Ethical Debate on Artificial Womb Technology*, 23.5 Am. J. Bioethics 110 (2023).

7
Eliot Freidson, Profession of Medicine: A Study of the Sociology of Applied Knowledge (University of Chicago Press, 1988); Talcott Parsons, The Social System (Routledge, 2013).

8 Arianne Shahvisi, *Conscientious Objection: A Morally Insupportable Misuse of Authority*, 13.2 Clin. Ethics 82 (2018).

9 For a further central theme, see Victoria Adkins, *Location, Location, Location: The Approach of Healthcare Professionals in Defining the Artificially Gestated Entity*, 33.3 Med. Law. Rev fwaf035 (2025), which relates to how healthcare professionals define the artificially gestated entity. A further theme will be reported in a future publication regarding healthcare professionals’ responses to artificial placenta devices being used beyond medical need.

10 Reference to the fetus here is to the entity undergoing human pregnancy prior to any transfer to an artificial placenta device. See Adkins, *supra* note 9, for an analysis of the participants’ perspectives on the status of an entity once it is within an artificial placenta device.

11 NHS, *Keeping Well in Pregnancy,*  https://www.nhs.uk/pregnancy/keeping-well/ (accessed Aug. 28, 2025)

12 Birmingham Health Partners, *Healthy Mum, Healthy Baby, Healthy Future,*  https://www.birminghamhealthpartners.co.uk/wp-content/uploads/2022/05/Final-Healthy-Mum-Healthy-Baby-Healthy-Future-Report-AW_Accessible-PDF-REDUCED-FILE-SIZE.pdf (accessed Aug, 29, 2025); Ethel D. Weld, Theodore C. Bailey and Catriona Waitt, *Ethical Issues in Therapeutic Use and Research in Pregnant and Breastfeeding Women*, 88.1 Br. J. Clin. Pharmacol. 7 (2022); Mary C. Blehar, Catherine Spong, Christine Grady, Sara F. Goldkind, Leyla Sahin, and Janine A. Clayton, *Enrolling Pregnant Women: Issues in Clinical Research,* 23.1 Womens Health Issues e39 (2013).

13 Anne D. Lyerly, Lisa M. Mitchell, Elizabeth M. Armstrong, Lisa H. Harris, Rebecca Kukla, Miriam Kupperman, and Margaret O. Little, *Risk and the Pregnant Body*, 4 Asian Bioeth. Rev. 367 (2009).

14 Giulia Cavaliere, *Gestation, Equality and Freedom: Ectogenesis as a Political Perspective* 46 J. Med. Ethics. 79 *(*2020).

15 MBRRACE-UK, *Maternal Mortality 2020–2022,*  https://www.npeu.ox.ac.uk/mbrrace-uk/data-brief/maternal-mortality-2020-2022#main-points (accessed Aug. 28, 2025).

16 Sophie L. Schott, Faith Fletcher, Alice Story, and April Adams, *Addressing or Reinforcing Injustice? Artificial Amnion and Placenta Technology, Loss-Sensitive Care and Racial Inequities in Preterm Birth*, 50.5 J. Med. Ethics 316 (2024); Elizabeth Chloe Romanis and Claire Horn, *Artificial Wombs and the Ectogenesis Conversation: A Misplaced Focus? Technology, Abortion, and Reproductive Freedom* 13.2 Ijfab 174, 247 (2020).

17 Peter Conrad, *Medicalization and Social Control*, 18.1 Annu. Rev. Sociol. 209 (1992).

18 Helen MacKenzie Bryers and Edwin Van Teijlingen, *Risk, Theory, Social and Medical Models: A Critical Analysis of the Concept of Risk in Maternity Care*, 26.5 Midwifery 488 (2010).

19
*Id*.

20
Emily Jackson, Regulating Reproduction: Law, Technology And Autonomy (Bloomsbury, 2001); Elizabeth Armstrong, Conceiving Risk, Bearing Responsibility: Fetal Alcohol Syndrome And The Diagnosis Of Moral Disorder (John Hopkins University Press 2003).

21 In February 2016, it was reported that maternal mortality rates, as well as stillbirth and neonatal mortality rates, have significantly reduced since 2003 (National Maternity Review, *Better Births: Improving Outcomes of Maternity Services in England*, https://www.england.nhs.uk/publication/better-births-improving-outcomes-of-maternity-services-in-england-a-five-year-forward-view-for-maternity-care/ (accessed Aug. 28, 2025).

22 Joan Liaschenko, Debra DeBruin, and Mary F. Marshall, *The Two-Patient Framework for Research during Pregnancy: A Critique and a Better Way Forward,* 11.5 Am. J. Bioeth. 66 (2011).

23 Adkins, *supra* note 4, at 239–240.

24 Lyerly and others, *supra* note 13, at 371.

25
*Id.* at 370–371.

26 Jackson, *supra* note 20, at 119–131.

27 Gregory Pence, *What’s So Good About Natural Motherhood? (in Praise of Unnatural Gestation)* in Ectogenesis: Artificial Womb Technology And The Future Of Reproduction (S. Gelfand and J. Shook, eds, 2006), 82.

28 Romanis, *supra* note 4; Elizabeth Chloe Romanis, *Artificial Womb Technology and the Significance of Birth: Why Gestatelings Are Not Newborns (or Fetuses),* 45 J. Med. Ethics 728 (2019).

29 Elselijn Kingma and Suki Finn, *Neonatal Incubator or Artificial Womb? Distinguishing Ectogestation and Ectogenesis Using the Metaphysics of Pregnancy*, 34.4 Bioethics 354, 360 (2020).

30
*Id*.

31 Nick Colgrove, *Subjects of Ectogenesis: Are ‘Gestatelings’ Fetuses, Newborns or Neither?*, 45.11 J. Med. Ethics. 723, 724 (2019).

32 Haruo Usuda, S. Watanabe, Hanita T., M. Saito, S. Sato, H. Ikeda, Y. Kumagai, M.C. Choolani and M.W. Kemp, *Artificial Placenta Technology: History, Potential and Perception,* 141 Placenta 10, 15 (2023).

33 Adkins, *supra* note 9.

34 Claire Horn and Elizabeth Chloe Romanis, *Establishing Boundaries for Speculation about Artificial Wombs, Ectogenesis, Gender, and the Gestating Body,* in A Jurisprudence of the Body (C. Dietz, M. Travi and M. Thomson, eds, 2020).

35 Victoria Adkins, *The Warnock Report and Partial Ectogestation: Retracing the Past to Step into the Future*, 31.3 Med. Law Rev. 424 (2023).

36 Maureen Sander-Staudt, *Of Machine Born? A Feminist Assessment of Ectogenesis and Artificial Wombs,* in Ectogenesis: Artificial Womb Technology and the Future of Reproduction (S. Gelfand and J. Shook, eds, 2006).

37
Evie Kendal, Equal Opportunity and the Case for State Sponsored Ectogenesis (Springer, 2015).

38 Peter Singer and Dianne Wells, *Ectogenesis,* in Ectogenesis: Artificial Womb Technology and the Future of Reproduction (S. Gelfand and J. Shook, eds, 2006).

39 Sander-Staudt, *supra* note 36.

40 Singer and Wells, *supra* note 38.

41 Kenneth J. Arrow and Anthony C. Fisher, *Environmental Preservation, Uncertainty, and Irreversibility*, in Economics of Natural and Environmental Resources (V. Smith, ed, 2013); Verna Jans, Wybo Dondorp, Sebastiaan Mastenbroek, Heidi Mertes, Guido Pennings, Hubert Smeets, and Guido de Wert, *Between Innovation and Precaution: How Did Offspring Safety Considerations Play a Role in Strategies of Introducing New Reproductive Techniques?*, 2020.2 Hum. Reprod. Open hoaa003 (2020).

42 Per Sandin, *Dimensions of the Precautionary Principle,* 5.5 Hum. Ecol. Risk Assess. 889 (1999).

43 Bryers and Teijlingen, *supra* note 18.

44 Freidson, *supra* note 7; Parson *supra* note 7.

45
Sally Sheldon, Beyond Control: Medical Power and Abortion Law (Pluto Press 1997).

46
Alan Bryman, Social Research Methods (Oxford University Press, 2016).

47 Partridge and others, *supra* note 1.

48 Adkins, *supra* note 9.

49
*Id.*

50 Virginia Braun and Victoria Clarke, *Using Thematic Analysis in Psychology*, 3.2 Qual. Res. Psychol. 77 (2006); Virginia Braun and Victoria Clarke, Successful Qualitative Research: A Practical Guide (SAGE, 2013); Virginia Braun And Victoria Clarke, Thematic Analysis: A Practical Guide (SAGE, 2022).

51 Braun and Clarke, 2006, *supra* note 50 at 79.

52 Braun and Clarke, 2022, *supra* note 50.

53 Braun and Clarke, 2022, *supra* note 50 at 12–22.

54 Virginia Braun and Victoria Clarke, *Can I Use TA? Should I Use TA? Should I Not Use TA? Comparing Reflexive Thematic Analysis and Other Pattern-Based Qualitative Analytic Approaches*, 21.1 Couns, Psychother. Res. 37, at 39–40 (2021).

55 Braun and Clarke, 2006, *supra* note 50 at 83.

56
*Id.* at 84–85; Braun and Clarke, 2013, *supra* note 50 at 207–208.

57 Braun and Clarke, 2013, *supra* note 50 at 225.

58
*Id.* at 234–235.

59 Braun and Clarke, 2022, *supra* note 50 at 85–87.

60 see Kingma and Finn, *supra* note 29 and E. Verweij, Lien De Proost, Judith OEH van Laar, Lily Frank, Sylvia A. Obermann-Borstn, Marijn J. Vermeulen, Sophie van Baalen, M. Beatrijs van der Hout-van der Jagt, and Elselijn Kingma, *Ethical Development of Artificial Amniotic Sac and Placenta Technology: A Roadmap*, 9 Front. Pediatr. 793,308 (2021).

61 Kingma and Finn, *supra* note 29.

62 Sandin, *supra* note 42.

63 Sander-Staudt, *supra* note 36.

64 Sandra Rees, Richard Harding and David Walker, *An Adverse Intrauterine Environment: Implications for Injury and Altered Development of the Brain*, 26.1 Int. J. Dev. Neurosci. 3 (2008).

65 Oskovi Kaplan, Zeynep Asli and A. Seval Ozgu-Erdinc, *Prediction of Preterm Birth: Maternal Characteristics, Ultrasound Markers, and Biomarkers: An Updated Overview*, 2018.1 J. Pregnancy 8367571 (2018); Mathilde Letouzey, Laurence Foix-L’Hélias, Héloïse Torchin, Ayoub Mitha, Andrei S. Morgan, Jennifer Zeitlin, Gilles Kayem *et al*., *Cause of Preterm Birth and Late-Onset Sepsis in Very Preterm Infants: The EPIPAGE-2 Cohort Study*, 90.3 Pediatr. Res. 584 (2021).

66 British Association of Perinatal Medicine, *Perinatal Management of Extreme Preterm Birth before 27 Weeks of Gestation: A Framework for Practice*, https://hubble-live-assets.s3.amazonaws.com/bapm/attachment/file/182/Extreme_Preterm_28-11-19_FINAL.pdf (accessed July 30, 2024) at 13.

67 World Health Organization, *Born Too Soon: Decade of Action on Preterm Birth*, https://www.who.int/publications/i/item/9789240073890 (accessed Aug. 24, 2025).

68 Sander-Staudt, *supra* note 36.

69 Christine Rosen, *Why Not Artificial Wombs?,* 2003.3 The New Atlantis 67 (2003).

70 Hammond-Browning, *supra* note 4.

71 Singer and Wells, *supra* note 38.

72 Horn and Romanis, *supra* note 34.

73 A similar position was taken by Dutch parents and healthcare professionals in relation to the outcomes of clinical trials: de Boer and others, *supra* note 5.

74 Katie Gallagher, Narendra Aladangady and Neil Marlow, *The Attitudes of Neonatologists towards Extremely Preterm Infants: A Q Methodological Study*, 101.1 Arch. Dis. Child-Fetal 31 (2016).

75 Dorian Accoe, Clemence Van Ginneken, and Seppe Segers, *Speculation as an Argument: Artificial Placenta Technology, Clinical Translation, and the Ethical Debate about the Ethical Debate*, Monash Bioeth. 1, 7 (2025).

76 Verweij and others, *supra* note 60; M. van der Hout-van der Jag, Beatrijs, E. Joanne T. Verweij, Peter Andriessen, Willem P. de Boode, Arend F. Bos, Frank LM Delbressine, Alex J. Eggink *et al*., *Interprofessional Consensus Regarding Design Requirements for Liquid-Based Perinatal Life Support (PLS) Technology*, 9 Front. Pediatr. 793531 (2022).

77 Susan Golombok, Fiona MacCallum and Emma Goodman, *The ‘Test-Tube’ Generation: Parent–Child Relationships and the Psychological Well-Being of In Vitro Fertilization Children at Adolescence* 72.2 Child Dev. 599 (2001).

78 Accoe and others, *supra* note 75, at 8 (2025).

79
Ann Oakley, The Captured Womb; A History of the Medical Care of Pregnant Women 205 (Basil Blackwell Ltd, 1984).

80 Olga Najera, *Ethical Concerns for Assisted Reproductive Technologies*, 3.1 Dialogue and Nexus 19 (2016).

81 Nuffield Council on Bioethics, *Ideas about Naturalness in Public and Political Debates About Science, Technology and Medicine—Analysis Paper*, https://www.nuffieldbioethics.org/assets/pdfs/Naturalness-analysis-paper.pdf (accessed July 30, 2024).

82 Sarah Church and Merryn Ekberg, *Student Midwives’ Responses to Reproductive Ethics: A Qualitative Focus Group Approach Using Case Scenarios*, 29.8 Midwifery 895 (2013).

83 International Confederation of Midwives, *Philosophy and Model of Midwifery Care*, https://internationalmidwives.org/resources/philosophy-and-model-of-midwifery-care/ (accessed Dec. 15, 2024).

84 Anna Smajdor, *The Moral Imperative for Ectogenesis*, 16.3 Camb. Q. Healthc. Ethic. 336 (2007); Kendal, *supra* note 37.

85 Anna Smajdor, *In Defense of Ectogenesis*, 21.1 Camb. Q. Healthc. Ethic. 90 (2012).

86 Peter Singer and Dianne Wells, *In Vitro Fertilisation: The Major Issues*, 9.4 J. Med. Ethics 192 (1983).

87 Mark Mercurio, *The EXTEND System for Extrauterine Support of Extremely Premature Neonates: Opportunity and Caution*, 84.6 Pediatr. Res. 795 (2018).

88 Singer and Wells, *supra* note 86.

89 Mercurio, *supra* note 87.

90 Richard Norman, *Interfering with Nature*, 13.1 J. Appl. Philos. 1 (1996).

91 Smajdor, 2007, *supra* note 84.

92 Emily McTernan, *Uterus Transplants and the Insufficient Value of Gestation*, 32.8 Bioethics 481, at 485 (2018).

93 Verweij and others, *supra* note 60; van der Hout-van der Jag and others, *supra* note 76.

94 Bryers and Teijlingen, *supra* note 18.

95 Whether partial ectogestation would fall within the remit of this legislation is questionable—see Adkins, *supra* note 4.

96 See for example Anna Nelson, *Should Delivery by Partial Ectogenesis Be Available on Request of the Pregnant Person?*, 15.1 International Journal of Feminist Approaches to Bioethics 1 (2022). Whilst Nelson does not specifically refer to the technology being safe for foetuses, it may be assumed that this is what is meant since the success of the technology is currently being measured by the outcomes for the gestated entity in current research with lambs. This point is discussed in more detail in chapter seven. A similar caveat can be found in Vera Tripodi, *The Right and Unfair Aspects of Artificial Womb Technology*, in Being and Value in Technology (E. Terrone and V. Tripodi, eds, 2022) 106.

97 Drew Carter and Annette Braunack-Mayer, *The Appeal to Nature Implicit in Certain Restrictions on Public Funding for Assisted Reproductive Technology* 25.8 Bioethics 463 (2011).

98
*Id*. at 465.

99 Segers, Pennings and Mertes, *supra* note 4; Seppe Segers, *The Path toward Ectogenesis: Looking beyond the Technical Challenges*, 22.1 Bmc Med. Ethics 59.

100 Rosen, *supra* note 69; Sander-Staudt, *supra* note 36; Elizabeth Chloe Romanis, Dunja Begović, Margot R. Brazier, and Alexandra Katherine Mullock, *Reviewing the Womb,* 47.12 J. Med. Ethics 820 (2021).

101 Margaret Ginoza, and Rosario Isasi, *Regulating Preimplantation Genetic Testing across the World: A Comparison of International Policy and Ethical Perspectives,* 10.5 Csh Perspect. Med. a036681 (2020).

102
Sarah Franklin and Emily Jackson, The 14 Day Rule and Human Embryo Research: A Sociology of Biological Translation (Taylor & Francis, 2024).

103 Richard Johanson, Mary Newburn and Alison Macfarlane, *Has the Medicalisation of Childbirth Gone Too Far?,* 324.7342 Bmj 892 (2002).

104 Tammy S. Harpel, *Fear of the Unknown: Ultrasound and Anxiety about Fetal Health*, 12.3 Health 295 (2008); Raphaël P. Hammer and Claudine Burton-Jeangros, *Tensions around Risks in Pregnancy: A Typology of Women’s Experiences of Surveillance Medicine*, 93 Soc. Sci. Med. 55 (2013).

105 Stephanie Meredith, Scotti Brackett, Keith M. Diaz, Kathleen G. Freeman et al., *Recommendations to Improve the Patient Experience and Avoid Bias When Prenatal Screening/Testing*, 16.2 Disabil. Health J. 101401 (2023); Felicity Boardman and Gareth Thomas, *Expressivist Objections to Prenatal Screening and Testing: Perceptions of People Living with Disability*, 45.6 Sociol. Health Illn. 1233 (2023).

106 Rosemarie Garland-Thomson, *The Case for Conserving Disability*, 9.3 J. Bioeth. Inq. 339, 339–340 (2012).

107 I am grateful to an anonymous reviewer for raising this point.

108 Garland-Thomson, *supra* note 106.

109 Accoe and others, *supra* note 75, at 7.

110 Claire Blakeley, Debbie M. Smith, Edward D. Johnstone, and Anja Wittkowski, *Parental Decision-Making Following a Prenatal Diagnosis That is Lethal, Life-Limiting, or Has Long Term Implications for the Future Child and Family: A Meta-Synthesis of Qualitative Literature*, 20.1 Bmc Med. Eth. 56 (2019).

111 Shahvisi, *supra* note 8, at 83; Sarah Devaney and Søren Holm, *The Transmutation of Deference in Medicine: An Ethico-Legal Perspective*, 26.2 Med. Law Rev. 202 (2018).

112 Amelia DeFalco and Luna Dolezal, *Artificial Wombs and the Politics of Disability Futures*, https://itdfproject.org/artificial-wombs-and-the-politics-of-disability-futures/#:~:text=Kafer‘s%20reflections%20on%20disability%20futures,lives%20are%20valued%20within%20society. (accessed Aug. 24, 2025).

113 Accoe and others, *supra* note 75, at 7.

114 Romanis, 2019, *supra* note 28, at 729; Elizabeth Chloe Romanis and Victoria Adkins, *Artificial Placentas, Pregnancy Loss and Loss-Sensitive Care,* 50.5 J. Med. Ethics 299 (2024); Hannah Carpenter, Georgia Loutrianakis, Peyton Baker, Tiffany Bystra, and Lisa Campo-Engelstein, *Procreative Loss without Pregnancy Loss: The Limitations of Fetal-Centric Conceptions of Pregnancy*, 50.5 J. Med. Ethics 310 (2024); Schott, *supra* note 21.

115 Romanis and Adkins, *supra* note 114.

116 Laura M. Purdy, *Are Pregnant Women Fetal Containers?,* 4.4 Bioethics 273 (1990); Elselijn Kingma, *Were You a Part of Your Mother?*, 128.511 Mind 609 (2019); Jackson, *supra* note 20; Emily Jackson, *Degendering Reproduction?*, 16.3 Med. Law Rev. 346 (2008).

117 Jackson, *supra* note 20; Armstrong, *supra* note 20.

118 E. Albert Reece, *First Trimester Prenatal Diagnosis: Embryoscopy and Fetoscopy*, 23.5 Semin. Perinatol. 424 (1999).

119 Frank A. Chervenak and Laurence B. McCullough, *Ethical Dimensions of the Fetus as a Patient*, 43 Best Pract. Res. Cl. Ob. 2 (2017).

120 Anne D. Lyerly, Margaret O. Little, and Ruth R. Faden, *A Critique of the Fetus as Patient*, 8.7 The Am. J. Bioethics 42 (2008).

121 Pence, *supra* note 27.

122 Singer and Wells, *supra* note 38; Bruce P. Blackshaw, Bruce and Daniel Rodger, *Ectogenesis and the Case against the Right to the Death of the Foetus*, 33.1 Bioethics 76 (2019).

123 Segers, Pennings, and Mertes, *supra* note 4.

124 Hammond-Browning, *supra* note 4.

125 Romanis and others, *supra* note 100.

126 See Adkins, *supra* note 9. A future publication, drawing upon a further theme from this study will also illustrates the consistent fetal focus of the participants in this study throughout the interviews, despite questions asked about pregnant individuals.

127 Romanis and Adkins, *supra* note 114.

128
Pam Lowe, Reproductive Health and Maternal Sacrifice (Palgrave McMillan, 2016); Zaina Mahmoud and Elizabeth Chloe Romanis, *On Gestation and Motherhood*, 31.1 Med. Law Rev. 109 (2023).

129 Jackson, *supra* note 20.

130 Sylvia Burrow, *Reproductive Autonomy and Reproductive Technology*, 16.1 Techne: Res. Philos. Technol. (2012); Romanis and others, *supra* note 100.

131 John Harris and Søren Holm, *Extending Human Lifespan and the Precautionary Paradox*, 27.3 J. Med. Philos. 355 (2002).

132 Ruud H.J. ter Meulen, *The Ethical Basis of the Precautionary Principle in Health Care Decision Making*, 207.2 toxicol. Appl. Pharm. 663 (2005).

